# Robotic axial lower leg testing: repeatability and reproducibility

**DOI:** 10.1007/s00167-015-3768-4

**Published:** 2015-09-10

**Authors:** Thomas Branch, Shaun Stinton, Maya Sternberg, William Hutton, Frédéric Lavoie, Christian Guier, Philippe Neyret

**Affiliations:** University Orthopedics, Decatur, GA USA; ArthroMetrix, LLC, 441 Armour Place NE, Atlanta, GA 30324 USA; Quartiles Consulting, Atlanta, GA USA; Department of Orthopaedics, Emory University School of Medicine, Atlanta, GA USA; Hôpital Notre-Dame du CHUM, Montréal, Canada; San Francisco Sports Medicine and Orthopaedic Surgery, San Francisco, CA USA; Department of Orthopaedic Surgery, Centre Albert Trillat, Hôpital de la Croix-Rousse, Lyon, France

**Keywords:** Knee laxity, Manual examination, Robotic testing, Reliability, Rotational laxity

## Abstract

**Purpose:**

The purpose of this study was to determine the test–retest reliability and the repeatability over multiple days of a robotic testing device when used to measure laxity of the lower leg during a simulated dial test.

**Methods:**

Ten healthy subjects were evaluated using an instrumented robotic lower leg testing system over 4 days. Three testing cycles were performed each day. Each leg was rotated into external and then internal rotation by servomotors until a torque threshold of 5.65 N m was reached. Load–deformation curves were generated from torque and rotation data. Both average-measure and single-measure intraclass correlation coefficients (ICC) were compared across the curves. ICC scores were also compared for features of the curves including: maximum external rotation at −5.65 N m of torque, maximum internal rotation at 5.65 N m of torque, rotation at torque 0, compliance (slope of load–deformation curve) at torque 0, endpoint compliance in external rotation, endpoint compliance in internal rotation, and play at torque 0. Play at torque 0 was defined as the width of the hysteresis curve at torque 0.

**Results:**

Average-measure ICC scores and test–retest scores were >0.95 along the entire load–deformation curve except around zero torque. ICC scores at maximum internal and external rotation ranged from 0.87 to 0.99 across the left and right knees. ICC scores for the other features of the curves ranged from 0.61 to 0.98. The standard error of the mean ranged from 0.0497 to 1.1712.

**Conclusions:**

The robotic testing device in this study proved to be reliable for testing a subject multiple times both within the same day and over multiple days. These findings suggest that the device can provide a level of reliability in rotational testing that allows for clinical use of test results. Objective laxity data can improve consistency and accuracy in diagnosing knee injuries and may enable more effective treatment.

## Introduction

To date, in order to diagnose pathology within the knee, physical examination and manual palpation of the knee have been performed through a series of manoeuvres/tests. Examples of these tests include the Lachman–Trillat test, pivot shift test, varus and valgus stress tests, anterior and posterior drawer tests, and the dial test. Since practitioners of these tests can be trained differently, uniformity in diagnosis can be difficult [3–5, 7, 11, 13–15, 19].

The aforementioned tests were designed to test translational instability, rotational instability, or a combination of the two. Beginning in the late 1960s, rotational abnormalities of the knee became a focus for describing ligament damage [8, 9, 12, 18]. The terms anteromedial and anterolateral rotatory instability were coined to describe patterns of knee injury and guide decision-making for treatment. The dial test is one method used to characterize rotatory instability.

The dial test evolved from the posterolateral drawer test as described by Hughston et al. [6]. The dial test can be performed with the subject supine or prone. It tests for increased internal and external rotation in the affected leg at 30° and/or 90° of knee flexion compared with the opposite, supposedly healthy leg. When performing the test, the foot is dorsiflexed to limit the movement of the ankle/foot relative to the tibia in order to isolate tibial rotation. However, the motion between the tibia and the ankle/foot cannot be eliminated entirely. The system that is truly being tested by the practitioner is the entire lower extremity since the rotatory force is being applied through the foot of the subject with the laxity being measured at the knee. This system of the foot, ankle, and tibia comprises the distal “interface” of the knee to the ground.

The dial test is a subjective test, like any manual clinical knee examination, with the goal of identifying rotational laxity. Instrumented devices have been developed in an attempt to quantify rotational laxity objectively; however, variability in the applied force, strain rate, and the set-up of the lower extremity have resulted in increased measurement error over multiple days of testing [1, 10, 17, 20]. Unlike instrumented devices, a robotic testing device can precisely control the positioning of the lower leg and can apply force consistently from test to test and from day to day through the use of a motor. A robotic testing device can generate precise clinical load–deformation curves during axial rotational testing of the tibia/ankle/foot (or “lower leg”) which may reduce the measurement error associated with instrumented devices.

The purpose of this study was to determine the test–retest reliability and the repeatability over multiple days of a robotic testing device when used to measure laxity of the lower leg during a simulated dial test.

## Materials and methods

Ten subjects (seven males and three females) with no history of knee injury were recruited in Lyon, France. Subjects consented to participate in this prospective, level II study. IRB approval was not required at the institution at the time of the testing. The sample size requirement was determined as described by Bonnet [2]. A sample size of ten subjects with at least three replicates ensured a 95 % confidence interval width of 0.2 when planning for a reliability of 0.90. The average age of subjects was 36.4 years (22.9–51.0 years). The average height and weight of the subjects were 1.76 m and 72.4 kg. The subjects were evaluated using a robotic testing device (US Patent #s: 7,753,863—apparatus and method for evaluating ligaments; 8,753,294—apparatus and method for evaluating ligaments; 8,840,570—multi-section limb and ligament evaluation apparatus and associated methods for using same) on each of four successive days, with three tests carried out on each subject per day. Each subject was evaluated a minimum of 12 h apart, but no effort was made to evaluate the subjects at the same time of the day. Two separate robotic testing devices were used during these tests, and four of the authors were involved in setting up the subjects. Test–retest reliability and the repeatability over multiple days of testing were determined by including data from both devices and all four clinicians who set up the subjects. Inter-tester reliability was not reported separately. Both testing devices were calibrated using the same procedure, and all testers received the same training. The training consisted of a single day of setting up volunteers in the devices and running practice tests.

**Fig. 1:**
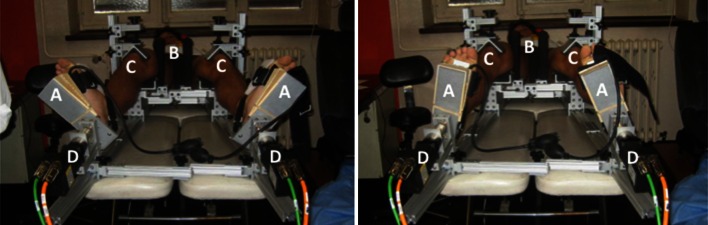
Robotic lower leg axial rotation system showing a patient whose feet are strapped into footplates (**a**), with both femurs stabilized using distal femoral posts (**b**), and both patellae locked into the trochlear grove with clamps (**c**) as torque is applied through the use of servomotors (**d**) during external rotation testing (l*eft*) and internal rotation testing (*right*)

Each subject had both extremities placed into the robotic testing device (Fig. 1). The knees were flexed to 30° using a goniometer with the distal femur resting on a pad 0.1 m proximal to the joint line. One medial and one lateral distal femoral post were used to position the distal femur and patella under the patellar clamp. A torque wrench was used to clamp these two posts around the femur with 4.52 N m of torque to reduce the perturbation of the femur that occurs with hip internal and external rotation. The patellar clamp was secured with 4.52 N m of torque, as measured by the torque wrench, which anchored the patella in the femoral groove in an anterior to posterior fashion. The heel was positioned within a padded “V,” and the forefoot was anchored to an L-shaped plate with a strap. To limit foot and ankle motion during rotational testing, the foot was maximally dorsiflexed using a small custom inflatable air bag at the level of the metatarsal heads.

After the patient was positioned in the robotic testing device, both upper legs were held fixed while the lower legs were rotated by footplates attached to and controlled by servomotors (Baldor Electric Company, Fort Smith, AR, USA). Each motor applied torque about the centre of rotation of the tibia (2.54 cm anterior to the heel at the plantar surface of the foot). The legs were initially rotated into external rotation. Rotation continued until peak external rotation torque equalled −5.65 N m. The motors then reversed direction and rotated until the peak internal rotation torque equalled 5.65 N m. This torque threshold was chosen to match the average level of torque applied by examiners who performed a manual dial test in pilot testing prior to this study. This level of torque is also similar to the levels used in previous studies which examined devices that simulate the dial test [1, 20]. Three rotation cycles were performed, and torque and rotation data were recorded. The three test cycles generally produced identical results, so only the third cycle was used for data analysis. Rotation of the leg was measured in degrees using the encoder count in the servomotor. Motor current was used to calculate torque applied to the leg. Accuracy was >0.01° and 0.001 N m of torque as determined by the specifications of the motor.

**Fig. 2:**
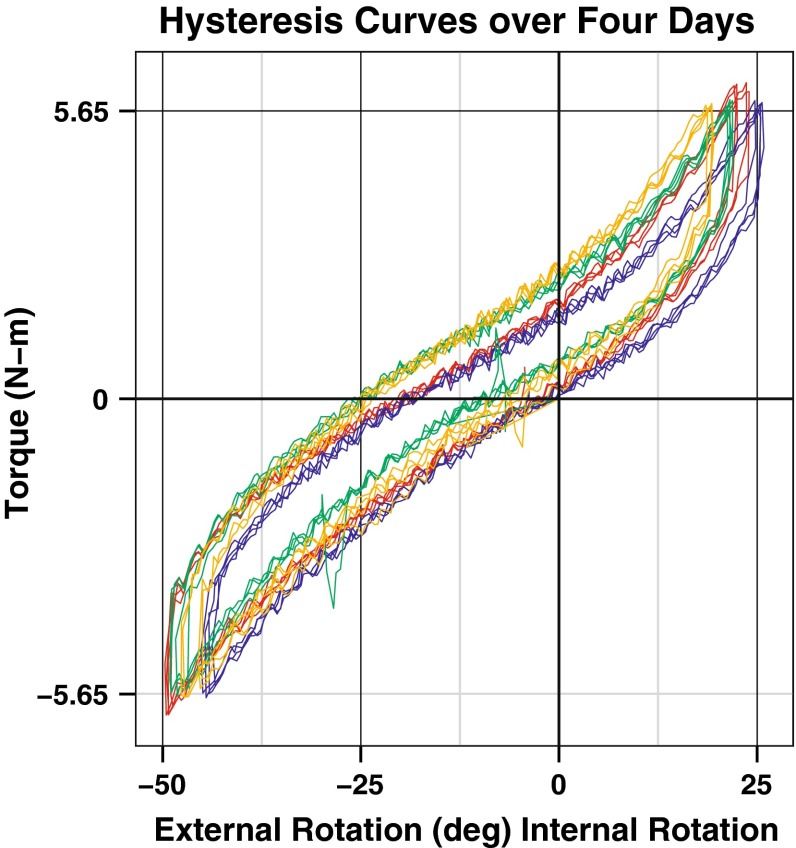
Hysteresis curves of all testing cycles over all days for the right leg in one subject. Each day is represented as a different colour with three cycles of overlapping data

**Fig. 3:**
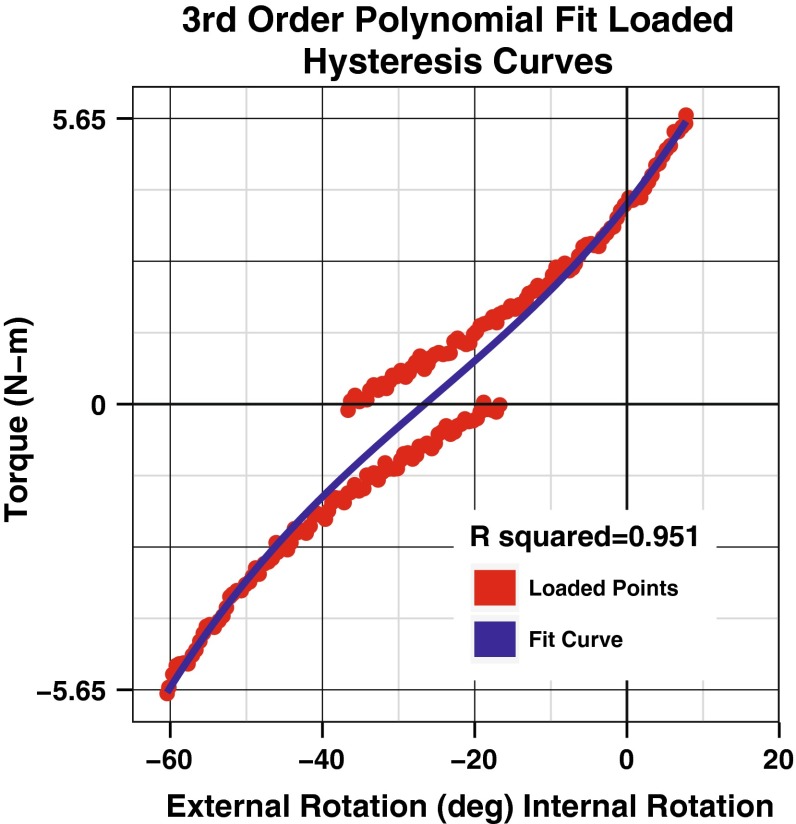
Loaded portion of a hysteresis curve is shown (in *red*) with a third-order polynomial fit for one cycle on a single day of testing

A custom MATLAB (MathWorks, Inc., Natick, MA) program was used for analysis of the torque and rotation data. Load–deformation curves were constructed for all 4 days with three cycles per day with torque on the *y*-axis and rotation on the *x*-axis (Fig. 2). Using the loaded portion of the hysteresis curve, a third-order polynomial fit of the data was used for analysis (Fig. 3). Once fitted, the curve was interpolated for a standard set of 500 points between −5.65 N m (external rotation) and +5.65 N m (internal rotation). This allowed for pointwise comparison of rotational position and torque across all subjects over all cycles and all days.

Intraclass correlation coefficients (ICC) were used as a measure of reliability in this study. ICC values were calculated using a MATLAB subroutine authored by Kevin Brownhill (Imaging Sciences, KCL, London kevin.brownhill@kcl.ac.uk) based on Shrout and Fleiss’s original paper [16]. For comparative purposes with previous literature, both average-measure ICC scores and single-measure ICC scores are reported. Both ICC scores for the full load–deformation curves and ICC scores for features of the curves were calculated. Features of the load–deformation curves that were examined included: maximum external rotation at −5.65 N m of torque, maximum internal rotation at 5.65 N m of torque, rotation at torque 0, compliance (slope of load–deformation curve) at torque 0, endpoint compliance in external rotation (ER), endpoint compliance in internal rotation (IR), and play at torque 0. Play at torque 0 was defined as the width of the hysteresis curve at torque 0.

A linear mixed model (LMM) was applied to estimate the magnitude of each source of measurement error and its relative contribution to the overall error. Measures of reliability and reproducibility were also obtained from the LMM. Through the use of a LMM, important sources of variation to the overall measurement process can be identified such as variation due to pure replication, different raters (or observers), days, instruments, locations, knees, or some combination thereof. To our knowledge, using linear mixed models with the ideas of generalizability theory has not been applied to reliability studies for knee instrumentation. The LMM was applied to analyse data for each of the features of the load–deformation curves. Three factors were considered: subjects, knees (left/right), and days (1–4). Since the goal is to generalize the results beyond only this sample of subjects, subject was a random effect. Knees were nested within subjects, since there is no reason to hypothesize that there is some consistent difference between the knees across individuals. By assuming all the effects are random, there were four possible sources of error: variability due to differences between subjects, variability due to differences between knees, variability due to measures taken on different days, and residual measurement error. Since the effects are nested, there were no interactions in the model.

## Results

**Fig. 4:**
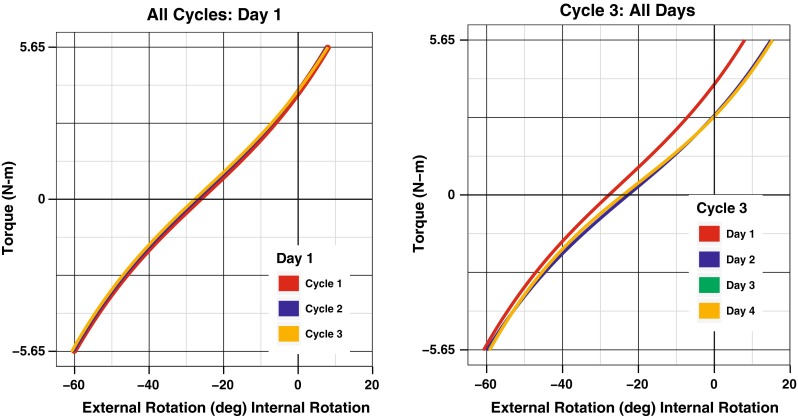
(*Left*) Load–deformation curves showing all cycles in 1 day in a single subject. (*Right*) Load–deformation curves showing the third cycle on each day in a single subject

**Fig. 5:**
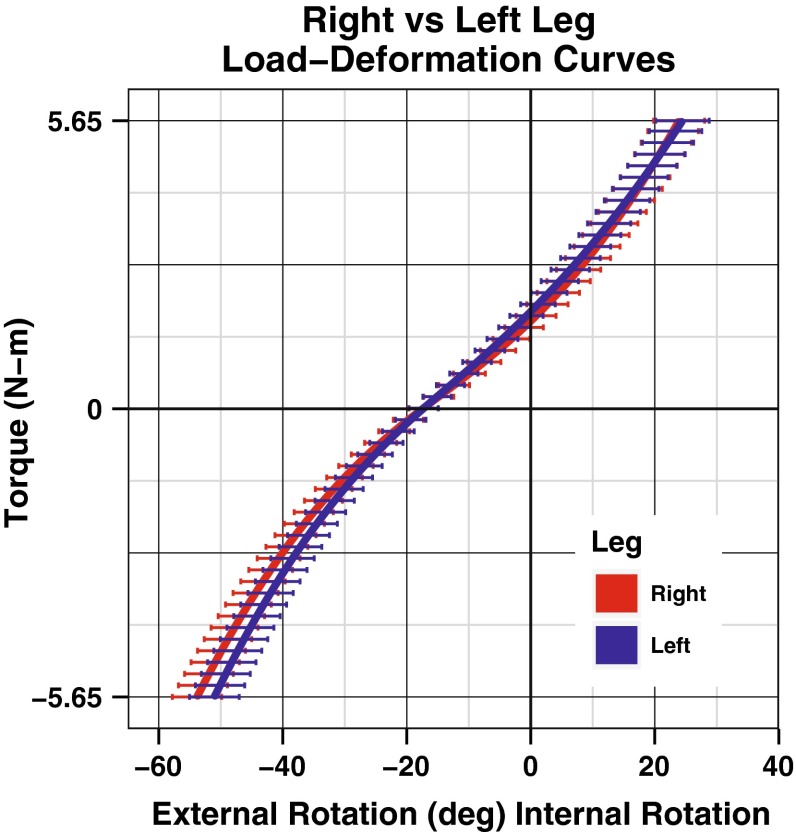
Mean load–deformation curves for the *right* and *left* legs of all subjects over all cycles and all days. The *error bars* represent standard error of the mean

Load–deformation curves showing all test cycles from a single day are shown in Fig. 4. Load–deformation curves of a single test cycle from four separate days are also shown in Fig. 4. A mean curve for both left and right knees with error bars representing the standard error of the mean (SEM) was constructed using the data from all days and all cycles (Fig. 5).

**Fig. 6:**
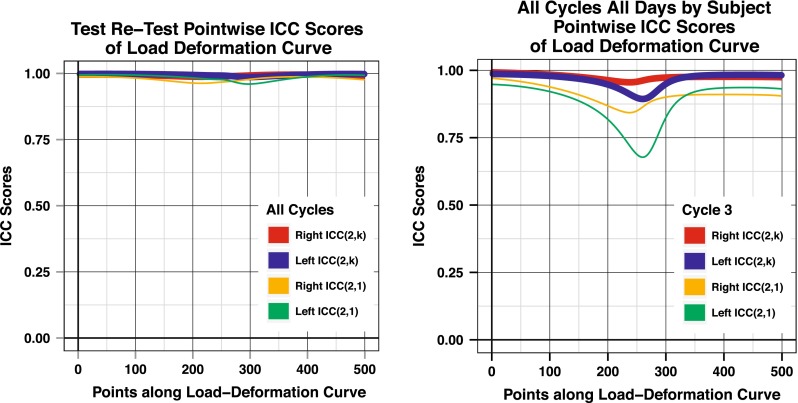
(*Left*) Pointwise test–retest ICC scores over all cycles in a single day. (*Right*) Pointwise ICC scores for all cycles and all days

Pointwise ICC scores along the entire load–deformation curves were calculated for all cycles within each day to evaluate test–retest reliability (Fig. 6). Similarly, pointwise ICC scores were calculated using all cycles over all days as a measure of reproducibility over days (Fig. 6).

The ICC scores of the features measured from the load–deformation curves are given in Table 1. Maximum ER, maximum IR, and slope at torque 0 have the highest reliability, and slope at maximum IR (endpoint compliance in IR) has the lowest reliability.

**Table 1:** Intraclass correlation coefficients (ICCs) for features of the load–deformation curves split by side and ICC type (single-measure ICC(2,1) and average-measure ICC(2,k))

Features	Feature ICC scores			
	Right ICC(2,k)	Right ICC(2,1)	Left ICC(2,k)	Left ICC(2,1)
Maximum ER	0.99	0.92	0.99	0.92
Maximum IR	0.99	0.87	0.99	0.91
Rotation at torque 0	0.97	0.75	0.99	0.85
Slope at torque 0	0.99	0.97	0.99	0.87
Slope IR	0.96	0.69	0.95	0.61
Slope ER	0.96	0.68	0.98	0.78
Play at torque 0	0.98	0.78	0.95	0.63

The results from the application of the linear mixed model are given in Tables 2 and 3. Table 2 summarizes the estimated components of variance, the standard error of measurement (square root of the residual error), the total standard deviation (square root of the sum of the variance components), test–retest reliability, day-to-day reproducibility, the overall mean, and the appropriate standard error of the mean from the linear mixed model.

**Table 2:** Results from the linear mixed model

	Variance components						Generalizability coefficients		Descriptive statistics	
Feature	Subject (σ^2^s)	Knee (σ^2^k)	Day (σ^2^d)	Residual (σ^2^e)	Standard error of measurement	Total SD	Test–retest reliability	Day-to-day reproducibility	Overall mean (both knees)	Standard error of the mean
Maximum ER	147.7	9.3	14.3	1.3	1.1	13.1	0.993	0.910	*−52.6°*	*3.9°*
Maximum IR	140.1	14.5	21.3	2.5	1.6	13.4	0.986	0.866	*23.9°*	*3.9°*
Rotation at Torque 0	42.8	7.9	14.2	1.8	1.3	8.2	0.973	0.760	*−17.4*	*2.2*
Slope at Torque 0	0.30	0.01	0.02	0.01	0.04	0.57	0.995	0.925	*1.3*	*0.17*
Slope IR	0.18	0.05	0.12	0.03	0.17	0.61	0.918	0.610	*2.0*	*0.15*
Slope ER	0.397	0.12	0.14	0.04	0.21	0.82	0.937	0.738	*1.8*	*0.21*
Play at Torque 0	53.8	7.0	17.6	11.9	3.4	9.5	0.868	0.673	*26.1*	*2.4*

Variance components describing the sources of error for each feature of the load–deformation curve are shown along with standard error or measurement and total standard deviation. There are four possible sources of error: variability due to differences between subjects, variability due to differences between knees, variability due to measures taken on different days, and residual measurement error. Generalizability coefficients describe the test–retest reliability and day-to-day reliability of each feature. Descriptive statistics (in italics) are also included showing the mean values and standard error of the mean for each feature. A negative value indicates external rotation (i.e. for rotation at torque 0, the average patient was in 17.4° of external rotation

**Table 3:** Relative contribution of each source of error and repeatability coefficients for features of the load–deformation curve

Feature	% due to subject	% due to knees	% due to day	% due to residual error	Repeatability coefficient r_s_
Maximum ER	85.6	5.4	8.3	0.7	3.48
Maximum IR	78.5	8.1	12.0	1.4	6.90
Rotation ar torque 0	64.2	11.8	21.3	2.7	4.95
Slope at torque 0	91.2	1.3	7.1	0.5	0.001
Slope IR	48.6	12.5	30.7	8.2	0.09
Slope ER	56.8	17.1	19.8	6.3	0.12
Play at torque 0	59.6	7.8	19.5	13.2	32.95

Features with larger relative contribution to error due to differences between subjects are more reliable measures. The repeatability coefficient describes the expected difference between two measurements for a given feature from the same subject and same knee. Maximum ER and IR describe the maximum rotation at 5.65 N m of torque in each direction. Slope IR and ER describe the slope of the endpoints of the load–deformation curves. Rotation at torque zero and slope at torque zero describe the rotational position and slope at the torque zero point on the load–deformation curve. Play at torque zero is the width of the hysteresis curve at the zero torque position

Table 3 provides estimates of the relative amount of variability due to each of the factors (subjects, knees, and days). Maximum ER, maximum IR, and slope at torque 0 each contribute significant subject to subject variability leading to high test–retest reliability and reproducibility coefficients. The feature with the least amount of variability explained due to differences between subjects and therefore a lower test–retest reliability is IR slope. A test of the components of each source of variability reveals that with the exception of ER slope, knee-to-knee variability is not significantly different from zero, suggesting that in this population among the features chosen there is little evidence that a difference exists between the knees. This shows that day-to-day effects within subjects are small compared with the differences between subjects, and that in non-injured persons, variability due to left and right knee differences tends to be along the order of magnitude as day-to-day differences or smaller. Table 3 reports the repeatability coefficient which estimates the expected difference between two measurements for a given feature from the same subject and knee. For example, in maximum ER, a pair of observations is expected to be within 3.5 degrees for 95 % of pairs of observations.

## Discussion

The most important finding in this study was that an instrumented dial test performed using a robotic knee testing device demonstrated excellent reliability over 4 days of testing by four clinicians using two testing devices. Pointwise test–retest ICC(2,1) scores were excellent (>0.96) over the cycles recorded during each evaluation, even near the neutral or torque 0 point. This allowed for the utilization of a single cycle as a representative of the rotational load–deformation curve for that knee without pointwise averaging of cycles. The pointwise day-to-day reliability as measured by ICC(2,1) scores was quite high approaching the endpoints; however, a decrease in scores exists near neutral or the torque 0 point. Features of the curves demonstrated good to excellent reliability scores under the same conditions. The reliability results from the current study compare favourable to results reported from previous studies investigating instrumented devices for measurement of rotatory knee laxity.

Almquist et al. [1] used the rotameter device to examine ten healthy subjects. Subjects were tested twice within a day for intra-day reliability, twice within a week for inter-day reliability, and twice on the same testing occasion by two different examiners for inter-tester reliability. Measurements were taken at 30°, 60°, and 90° of knee flexion and with torque application of 3, 6, and 9 N m at each level of knee flexion. The neutral position was taken as the “patients relaxed position”. Single-measure ICC(2,1) scores were reported as the measure of reliability. The within-day reliability of the rotameter varied from 0.59 to 0.94, with a reliability of 0.87 at 30° of knee flexion when 6 N m of torque was applied. For inter-day testing 1 week apart, the ICC(2,1) was 0.84 when testing at 30°. When another tester examined the patient at 30°, the ICC(2,1) was <0.70. In regard to endpoint compliance, the examiner is left to their own opinion as to “end point” feel.

Musahl et al. [10] described the reliability of a rotational laxity measurement device developed at the University of Pittsburgh in a study of four fresh-frozen cadaveric lower extremities. Two testers applied 6 N m of torque during five trials on each leg. This was repeated for knee flexion angles of 0°, 30°, 60°, and 90°. Single-measure ICC(2,1) scores were used to report intra-tester reliability and ranged from 0.94 to 0.99. Inter-tester reliability, as measured by average-measure ICC(2,k) scores, ranged from 0.95 to 0.99. Tsai et al. [20] performed further experiments using the same device in 11 male subjects with normal knees. Single-measure ICC(2,1) scores for intra-tester reliability within a single test session were over 0.95 for both 30° and 90° of knee flexion. The inter-tester reliability, as measured by average-measure ICC(2,k) scores, was 0.88 at 90° of flexion and 0.81 at 30° of flexion. The average-measure ICC(2,k) scores for test–retest reliability were 0.77 at 90° of knee flexion and were not reported for 30° of knee flexion.

In a study by Schultz et al. [17], the reliability of the Vermont Knee Laxity Device in measuring rotational laxity was determined in 20 healthy subjects. Each subject was tested twice within a 48-h period. Day-to-day reliability was calculated using ICC(2,k) scores. ICC(2,k) values for external rotation were 0.88 for right knees and 0.86 for left knees. ICC(2,k) values for internal rotation were 0.93 for right knees and 0.89 for left knees. The reliability of total rotation (internal + external) was 0.91 for right knees and 0.89 for left knees. No single-measure ICC(2,1) scores were reported.

It is important to note that ICC scores as a measure of inter-tester reliability need clarification. First, an ICC score as a measure of reliability represents the amount of “real” information in a test as opposed to “measurement error”. In this study, the two RKT devices are a consistent set of “raters” or “test devices”, but also a sample of these “raters” or “test devices”. As such, Shrout and Fleiss would recommend the use of an ICC(2,k) score for evaluation of reliability [16]. The value of *k* represents the number of tests that the ICC scores are measured against. If four tests are taken and the ICC score is measured against the average of these four tests, then the ICC(2,4) would be used. ICC(2,k) is a measure of the reliability of the mean of *k* tests. When *k* = 1, the ICC(2,1) score represents a single measure of agreement or absolute agreement. When *k* > 1, the ICC(2,k) score represents an average-measure agreement or a measure of the consistency of *k* tests. In the clinical setting, when *k* > 1, one would use an ICC(2,k) score when averaging a series of tests in the same clinical evaluation to represent the “final evaluation”. If only one test is used to represent an entire period of “evaluation” and, thus, represents the “final evaluation”, a single-measure ICC(2,1) score should be used. In this study, an ICC(2,1) score is a measure of the reliability of one single test during a time period.

Clinicians examine patients at single timepoints during clinic visits to gauge the progression of treatment. Rarely, do clinicians take weekly or monthly tests and average them for a result. Therefore, when it comes to medical device research, the guidelines of Shrout and Fleiss should be followed closely and the statistically stricter single-measure ICC(2,1) scores should be reported for instrumented knee examination devices. From Table 1, it is easy to see that average-measure ICC(2,k) scores are always higher than single-measure ICC(2,1) scores for agreement and can be misleading, particularly, if not reported as such.

By utilizing the data from the complete load–deformation curve, this is the first study that uses functional data analysis (FDA) for the evaluation of load–deformation curves in the knee. Standard statistical methods compare a feature or single point on the curve, such as maximum internal rotation. FDA utilizes statistical comparisons across the entire curve. A load–deformation curve has both magnitude and curve shape with the shape representing the relative stiffness along the curve. Since a single measurement (cycle) was reliable, ICC(2,k) scores were not used because averaging of load–deformation curves has an effect on both the magnitude and the shape of the curve. The effect on the shape of the curve can be unintentional if there are small offsets between each curve, but these offsets will have an effect on the stiffness across the curve. Therefore, in this study a single cycle, cycle three, was analysed to preserve its true shape.

In this study, the “neutral” position was defined as when the second toe was perpendicular to the ground during set-up using an electronic goniometer. Others have suggested that “neutral” should be taken at the “relaxed” position of the tibia determined by each individual patient. We agree with PS Walker that during rotational leg testing that there is a low stiffness region bounded by a high stiffness region at each extreme in the rotational load–deformation curve [21]. Unfortunately, testing results demonstrated that ICC(2,1) scores are less reliable at the centre part of the rotational load–deformation curve. Therefore, allowing the patient or examiner choose “neutral” has been less reliable. It is our opinion that there is no natural centre of the knee during unweighted rotational testing. Testing becomes significantly more reliable when set-up determines “neutral” position.

This study is not without limitations. Lower leg axial rotation was investigated rather than tibiofemoral axial rotation which can introduce motion between the foot/ankle and the tibia. However, the behaviour of the entire system of the lower extremities is important in the understanding of knee issues since that system determines the interaction between the knee and the ground. In this study, the foot and ankle were dorsiflexed maximally through the use of an air bag to minimize motion at these joints. Testing using the robotic knee testing device is also dependent on patient set-up within the machine. However, the device removes the effect of the rater (examiner) on reliability during testing. With appropriate set-up procedure training, the patient set-up effect would likely be less than any rater effect that would be experienced while using other testing systems. The reliability numbers were excellent in this study even with the use of two robotic devices and with four clinicians setting up subjects.

The robotic testing device in this study proved to be reliable for testing a subject multiple times both within the same day and over multiple days. These findings suggest that the device can provide a level of reliability in rotational testing that allows for clinical use of test results. In the future, objective laxity data could improve consistency and accuracy in diagnosing knee injuries and may enable more effective treatment.
